# Efficient algorithms for Longest Common Subsequence of two bucket orders to speed up pairwise genetic map comparison

**DOI:** 10.1371/journal.pone.0208838

**Published:** 2018-12-27

**Authors:** Lisa De Mattéo, Yan Holtz, Vincent Ranwez, Sèverine Bérard

**Affiliations:** 1 ISEM, Université de Montpellier, CNRS, IRD, EPHE, Montpellier, France; 2 Queensland Brain Institute, University of Queensland, Brisbane, Australia; 3 AGAP, Univ Montpellier, CIRAD, INRA, Montpellier SupAgro, Montpellier, France; Julius Kuhn-Institut, GERMANY

## Abstract

Genetic maps order genetic markers along chromosomes. They are, for instance, extensively used in marker-assisted selection to accelerate breeding programs. Even for the same species, people often have to deal with several alternative maps obtained using different ordering methods or different datasets, e.g. resulting from different segregating populations. Having efficient tools to identify the consistency and discrepancy of alternative maps is thus essential to facilitate genetic map comparisons. We propose to encode genetic maps by *bucket order*, a kind of order, which takes into account the blurred parts of the marker order while being an efficient data structure to achieve low complexity algorithms. The main result of this paper is an *O*(*n* log(*n*)) procedure to identify the largest agreements between two bucket orders of *n* elements, their *Longest Common Subsequence* (LCS), providing an efficient solution to highlight discrepancies between two genetic maps. The LCS of two maps, being the largest set of their collinear markers, is used as a building block to compute pairwise map congruence, to visually emphasize maker collinearity and in some scaffolding methods relying on genetic maps to improve genome assembly. As the LCS computation is a key subroutine of all these genetic map related tools, replacing the current LCS subroutine of those methods by ours –to do the exact same work but faster– could significantly speed up those methods without changing their accuracy. To ease such transition we provide all required algorithmic details in this self contained paper as well as an R package implementing them, named LCSLCIS, which is freely available at: https://github.com/holtzy/LCSLCIS.

## Introduction

Genetic maps represent the positioning of markers –e.g. genes, single nucleotide polymorphisms (SNPs), microsatellites– along chromosomes. The first genetic maps were produced as early as 1913 with the first insight in *Drosophila* chromosome organization proposed by A. H. Sturtevant [1]. The uses of genetic maps are diverse: from crop or livestock improvement, as they provide a way to link a genetic region to a trait of interest, to genome assembly, as they are used as a backbone for anchoring the contigs whose orientation and order on the chromosomes are unknown [2].

**Fig 1:**
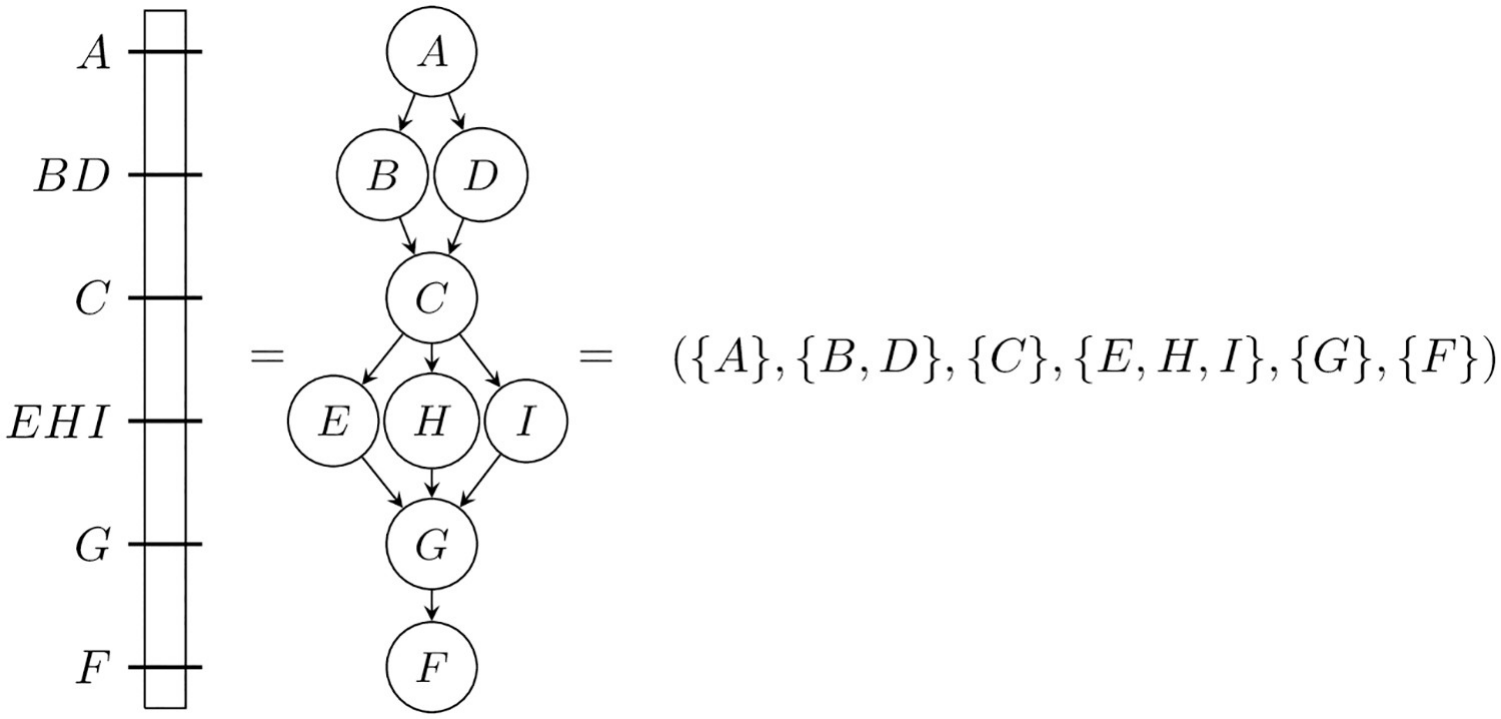
Simplified genetic map (left) and two different representations: A Directed Acyclic Graph (DAG) (middle) and a bucket order (right). In linkage maps, some markers may have the exact same position on a given map due to the absence of recombination events between them. In physical maps, this can happen when different genetic markers match at the same place.

Considering a linear chromosome, the corresponding genetic map should be a *total order* on all its markers. However, due to imprecisions, errors or inaccuracies of the techniques, it is usually a *partial order* on a subset of the markers. This is the case when the relative position of some markers cannot be inferred and they are put at the same position on the map, as illustrated on the left of Fig 1. We propose to model by a binary relation of order, namely a *bucket order*, maps where several markers are at the same position (Fig 1). A bucket order is a total order on *buckets*, each bucket containing elements which are incomparable [3]. Consequently, bucket orders are suitable structures for coding the genetic maps: they allow markers with an uncertain relative order to be gathered in a bucket while preserving the global order information, namely the bucket sequence. Moreover, even for a single species, we are often faced with several different maps obtained using different input data (e.g. different segregating population, sequencing techniques etc.) and different techniques or softwares to build a map from those data. The differences come from the subsets of markers positioned on the map or from their order. Recent works [4–6] show that it is possible, by comparing these different maps, to propose a richer and more reliable synthesis than what is obtained by a single approach.

This article focuses on identifying the largest subset of congruent information shared by two maps by identifying their *Longest Common Subsequence (LCS)*. In the genetic map framework, the LCS corresponds to the largest set of collinear markers, i.e. the largest set of markers that appears in the same order in the two compared maps. The LCS hence plays a key role in map comparison, as emphasized by ALLMAP authors: “Collinearity, defined as the arrangement of one sequence in the same linear order as another sequence, is one of the most important criteria in evaluating map concordance and evolutionary relatedness” [6]. Individual maps can be compared based on their correlation coefficients, for marker interval distance and for marker order, based on their LCS using the qualV package [7] as done for comparing switchgrass maps across studies [8, 9]. A visual representation of pairwise map collinearity is very helpful and several tools, such as MCScanX [10], VGSC [11] and the genetic map comparator [12], provide the so called “dual synteny plot representation”. This representation draws the two maps side by side and traces a line between their common markers. Identifying the LCS allows to highlight map collinearity by using different colors for linking markers that are part of the LCS and those that are not.

The LCS problem is encountered in many contexts, such as file comparison (e.g. unix diff command), computational linguistic analysis (e.g. [13]) or bioinformatics (e.g. sequence comparison and genome compression [14]) and has thus been extensively studied in computer science [15–17]. Finding a LCS for multiple input sequences has been proved NP-hard [18], while its pairwise counterpart is polynomial and often used in comparative genomics [19–21]. But as far as we know, the LCS problem has never been defined on bucket orders. From here on, we always refer to the pairwise LCS problem. The first step is to precisely define the notion of common subsequence on bucket orders. The adaptation is not so obvious and we propose two definitions relying on the linear extensions of bucket orders; one, called *Longest Common Induced Subsequence* (LCIS), being stricter than the other, that is simply called *Longest Common Subsequence* (LCS). The aim is that LC(I)S captures as much as possible of the consensual information contained in the input maps.

We demonstrate in this paper that we can compute LC(I)S on bucket orders with algorithms similar to the classical ones, once an adequate pre-treatment, that we called *homogenization*, has been performed. The consequence is that the search of the LCS then depends on the number of (homogenized) buckets rather than on the number of markers they contain. This could result in a significant performance improvement for genetic maps where several hundreds of markers can be in total linkage disequilibrium, hence in the same bucket. Homogenization is a kind of *partition refinement* technique largely used in efficient algorithms on finite automata, string sorting or graphs [22]. Usually, such algorithms run iteratively by splitting the current partition according to a subset of elements called the *pivot*. In our case, we homogenize one order/partition by the other order/partition using at the same time all its buckets as pivots. Our procedure leads to a simpler algorithm than [22] while achieving the same time complexity. Fagin *et al*. [23] defined a procedure similar to homogenization on bucket orders but to our knowledge they don’t provide algorithmic detail to produce it nor study its properties.

You will see that the organization of our paper does not follow the standard IMRaD format (Introduction, Methods, Results, and Discussion) as this is not well adapted for a methodological paper such as this one. In the next section, we present bucket orders and propose two definitions of longest common subsequence for bucket orders (LCS and LCIS). Then, we give details of procedure of homogenization used to allow bucket orders to behave like total orders in terms of time complexity. In the following section, we prove that searching for the LC(I)S of two bucket orders gives the same results as searching for the LC(I)S of their homogenized counterparts. Afterwards, we describe the algorithm which solves the problem, and give a variant to achieve the *O*(*n* log(*n*)) time complexity. Finally, in the last section, we briefly show an application of this work in the framework of dual synteny plots and we use simulated map datasets of various sizes to demonstrate the gain in speed thanks to our optimized LCS routine.

## Definitions and notations

In agronomy, marker-assisted selection strongly relies on genetic maps to accelerate breeding programs, see for instance [24]. Genetic maps provide an organization of marker along (fragment of) chromosomes. Each marker (SNPs, microsat etc.) is present at most once per genome to be useful for breeding programs. The ordering of those markers can, for instance, be deduced via the study of their linkage disequilibrium in a segregating population. Linkage disequilibrium basically reflects the fact that the value of two markers are not independent. When there is a complete linkage disequilibrium among a set of markers their dependence is total (for any individual of the population, knowing the value of one of those marker is enough to know the value of all other marker of this set). More generally, the higher the dependence between marker values, the closer the markers—since this dependence is related to the number of recombination events that have taken place in the population between those markers (e.g. [24]). Each (fragment) of chromosome of such a map can conveniently be represented by a sequence of bucket of markers, precedence in the sequence reflects precedence on the map while being in the same bucket reflects the fact that we have no clue about the relative position of those markers along this map. As marker are unique, each element/marker appears only once in this bucket sequence. Comparing the marker ordering of one (fragment of) chromosome proposed by two distinct genetic maps can thus be done by comparing the two equivalent bucket orders. Note that though this representation can be extended to store the distance between consecutive buckets of the sequence, we ignore here this information as it is irrelevant for the identification of the LC(I)S.

This section provides a more formal definition of bucket orders introduced above as well as an explicit definition of the LC(I)S on bucket orders. These definitions follow the order classification and notations used in [3].

A *binary relation R* on a domain D is a subset of D×D; here we denote *x* ≺_*R*_ *y* the fact that (*x*, *y*) ∈ *R*.

A binary relation *σ* is a *strict partial order* on D if, and only if, *σ* is:

- *irreflexive*: $\forall x\in D\;x{⊀}_{\sigma }x$;
- *asymmetric*: $\forall x,y\in Dx{≺}_{\sigma }y\Rightarrow y{⊀}_{\sigma }x$;
- *transitive*: ∀x,y,z∈D (*x* ≺_*σ*_ *y* and *y* ≺_*σ*_ *z*) ⇒ *x* ≺_*σ*_ *z*.

Two elements x,y∈D of a partial order *σ* are incomparable (*x* ≹_*σ*_ *y*) when neither *x* ≺_*σ*_ *y* nor *y* ≺_*σ*_ *x*.

**Definition 1** (Bucket order). A strict partial order *π* on D is a *bucket order* if, and only if, *π* is *negatively transitive*, i.e. ∀x,y,z∈D (*x* ⊀_*π*_ *z* and *z* ⊀_*π*_ *y*) ⇒ *x* ⊀_*π*_ *y*. It follows that D is partitioned into a sequence of *buckets* *B*_1_, …, *B*_*t*_ so that *x* ≺_*π*_ *y* ⇔ (*x* ∈ *B*_*i*_, *y* ∈ *B*_*j*_ and *i* < *j*). In a bucket order, elements are incomparable if, and only if, they belong to the same *bucket*. We denote by |*B*_*i*_| the number of elements in bucket *B*_*i*_.

For example, *π*_1_ = ({*k*}, {*a*, *b*}, {*l*, *c*}, {*d*, *e*, *f*}, {*i*, *j*}, {*g*, *h*}) is a bucket order on the domain $D_{1}=\left\{a,b,c,d,e,f,g,h,i,j,k,l\right\}$, which contains 6 buckets (for instance *B*_1_ = {*k*}, |*B*_4_| = 3), and where $a{≹}_{\pi_{1}}b$ while $a{≺}_{\pi_{1}}c$. A genetic map can easily be modelled by bucket orders on D, where D is the set of its markers.

Do not confuse bucket orders with indeterminate strings (also known as degenerate string) which are strings involving uncertainty and consist of nonempty subsets of letters over an alphabet Σ [25, 26]. In such strings the same character may appear in several subsets while it is not the case in bucket orders.

A *total order* *τ* is a *complete* partial order, that is ∀x,y∈D and *x* ≠ *y*, either *x* ≺_*τ*_ *y* or *y* ≺_*τ*_ *x*. In other words, all *τ* elements are comparable and *τ* is a permutation of the elements of D. Note that a total order can hence be seen as a bucket order $B_{1},\ldots ,B_{\left|D\right|}$ with all its buckets of size 1 or, alternatively, as a sequence of elements of D.

The definition of Common Subsequences of bucket orders relies obviously on the definition of a Subsequence of a bucket order *π*. We choose to define the latter as a subsequence of any total order compatible with *π*, more formally:

**Definition 2** (Bucket order subsequence). A *subsequence of a bucket order σ* on D is a sequence *s* = (*e*_1_, *e*_2_, …, *e*_*l*_) so that, ∀1 < *i* < *j* < *l*, $e_{i}\in D$ and either *e*_*i*_ ≺_*σ*_ *e*_*j*_ or $e_{i}{≹}_{\sigma }e_{j}$. We denote by subsequence(*σ*) the set of those subsequences.

**Definition 3** (Bucket order Common Subsequence and LCS). A *common subsequence* of two bucket orders *σ*_1_ on $D_{1}$ and *σ*_2_ on $D_{2}$ is a sequence *s* = (*e*_1_, *e*_2_, …, *e*_*l*_) of elements of $D=D_{1}\cap D_{2}$ so that: ∀1 < *i* < *j* < *l*

1. $e_{i}{≺}_{\sigma_{1}}e_{j}$ or $e_{i}{≹}_{\sigma_{1}}e_{j}$     i.e., *s* ∈ subsequence(*σ*_1_)
2. **and** $e_{i}{≺}_{\sigma_{2}}e_{j}$ or $e_{i}{≹}_{\sigma_{2}}e_{j}$   i.e., *s* ∈ subsequence(*σ*_2_)

The length of *s* is its number of elements, that is to say *l*. A common subsequence of maximum length is called a *Longest Common Subsequence (LCS)*.

Given two bucket orders *σ*_1_ and *σ*_2_ we denote subsequence(*σ*_1_, *σ*_2_) the set of their common subsequences. Let *π*_1_ = ({*k*}, {*a*, *b*}, {*l*, *c*}, {*d*, *e*, *f*}, {*i*, *j*}, {*g*, *h*}) and *π*_2_ = ({*g*, *h*}, {*c*, *d*, *e*, *f*}, {*m*, *q*}, {*r*, *a*}, {*b*, *n*}, {*o*, *p*, *l*}) be two bucket orders. The set of the LCS of *π*_1_ and *π*_2_ is {(*c*, *d*, *e*, *f*), (*c*, *d*, *f*, *e*), (*c*, *e*, *d*, *f*), (*c*, *e*, *f*, *d*), (*c*, *f*, *d*, *e*), (*c*, *f*, *e*, *d*)} and the length of their LCS is 4.

A common subsequence of two bucket orders *σ*_1_ and *σ*_2_ may arbitrarily arrange elements that are incomparable in both orders (i.e., such that $e_{i}{≹}_{\sigma_{1}}e_{j}$ and $e_{i}{≹}_{\sigma_{2}}e_{j}$), *e.g*. elements *d*, *e* and *f* in LCS of *π*_1_ and *π*_2_. In the context of genetic map comparison, one consequence is that an LCS of two maps may order two elements while no input map does. This is the motivation for the following definition, which is stricter than the previous one.

**Definition 4** (Bucket order Common Induced Subsequence and LCIS). A *common induced subsequence* of two bucket orders *σ*_1_ on $D_{1}$ and *σ*_2_ on $D_{2}$ is a sequence *s* = (*e*_1_, *e*_2_, …, *e*_*l*_) of elements of $D=D_{1}\cap D_{2}$ so that:

1. *s* ∈ subsequence(*σ*_1_, *σ*_2_) **and**
2. ∀1 < *i* < *j* < *l*:

(a) either $e_{i}{≺}_{\sigma_{1}}e_{j}$ and $e_{j}{⊀}_{\sigma_{2}}e_{i}$ **or** (b) $e_{i}{≺}_{\sigma_{2}}e_{j}$ and $e_{j}{⊀}_{\sigma_{1}}e_{i}$

The length of *s* is its number of elements, that is to say *l*. A common induced subsequence of maximum length is called a *Longest Common Induced Subsequence (LCIS)*.

Note that the ‘*and*’ parts of the 2^*nd*^ condition are implied by the fact that *s* ∈ subsequence(*σ*_1_, *σ*_2_) and are just useful reminders. Note also that an LCIS cannot contain several elements located in the same bucket in *σ*_1_ and in the same bucket in *σ*_2_ as they are incomparable in the two orders.

Given two bucket orders *σ*_1_ and *σ*_2_ we will denote as indSubsequence(*σ*_1_, *σ*_2_) the set of their induced subsequences. There is only one LCIS of *π*_1_ and *π*_2_: (*a*, *b*, *l*) and it is of length 3.

As far as we know, it is the first time that bucket orders, precise mathematical objects, are used to model genetic maps –including their blurred part. Moreover, we propose a rigorous extension of the classical problem of the LCS on these bucket orders, along with an alternative problem: the LCIS.

## Bucket order homogenization

This section introduces a preprocessing step, that we named *homogenization*, which refines two bucket orders so that those refined orders have only buckets that are either identical or with no common element. This preprocessing is the cornerstone of our efficient solution to find a LC(I)S of two bucket orders.

**Definition 5** (Homogenization of two bucket orders). Let $\sigma_{1}=\left(B_{1}^{1},\ldots ,B_{k_{1}}^{1}\right)$ be a bucket order on $D_{1}$ and $\sigma_{2}=\left(B_{1}^{2},\ldots ,B_{k_{2}}^{2}\right)$ be a bucket order on $D_{2}$. Let D be $D_{1}\cap D_{2}$, for each element e∈D. Let *pos*_1_(*e*) and *pos*_2_(*e*) denote the positions of the *bucket* containing *e* in the bucket sequence *σ*_1_ and *σ*_2_ respectively.

The *homogenization* of *σ*_1_ and *σ*_2_ associates to those orders the homogenized bucket orders $\sigma_{1}^{h}$ and $\sigma_{2}^{h}$ respectively, which are both defined on D and so that $\forall e,e^{\prime }\in D$:

- *e* and *e*′ belong to the same *bucket* of $\sigma_{1}^{h}$ (resp. $\sigma_{2}^{h}$) if, and only if, they are in the same bucket in both *σ*_1_ and *σ*_2_, *i.e*., if, and only if, *pos*_1_(*e*) = *pos*_1_(*e*′) and *pos*_2_(*e*) = *pos*_2_(*e*′);
- the *bucket* of $\sigma_{1}^{h}$ (resp. $\sigma_{2}^{h}$) containing *e* precedes the *bucket* of $\sigma_{1}^{h}$ (resp. $\sigma_{2}^{h}$) containing *e*′ if, and only if, *pos*_1_(*e*) < *pos*_1_(*e*′) or (*pos*_1_(*e*) = *pos*_1_(*e*′) and *pos*_2_(*e*) < *pos*_2_(*e*′)) (resp. *pos*_2_(*e*) < *pos*_2_(*e*′) or (*pos*_2_(*e*) = *pos*_2_(*e*′) and *pos*_1_(*e*) < *pos*_1_(*e*′))).

For example, let

*π*_1_ = ({*k*}, {*a*, *b*}, {*l*, *c*}, {*d*, *e*, *f*}, {*i*, *j*}, {*g*, *h*}) and

*π*_2_ = ({*g*, *h*}, {*c*, *d*, *e*, *f*}, {*m*, *q*}, {*r*, *a*}, {*b*, *n*}, {*o*, *p*, *l*}).

Their homogenized counterparts are

$\pi_{1}^{h}=\left(\left\{a\right\},\left\{b\right\},\left\{c\right\},\left\{l\right\},\left\{d,e,f\right\},\left\{g,h\right\}\right)$ and

$\pi_{2}^{h}=(\left\{g,h\right\},\left\{c\right\},\left\{d,e,f\right\},\left\{a\right\},$ {*b*}, {*l*}).

**Property 1**. Let *σ*_1_ be a bucket order on $D_{1}$, *σ*_2_ be a bucket order on $D_{2}$ and $\sigma_{1}^{h}$ and $\sigma_{2}^{h}$ be their respective homogenized bucket orders, then $\sigma_{1}^{h}$ and $\sigma_{2}^{h}$ have exactly the same buckets but not necessarily in the same order.

**Proof**. By contradiction. Let us assume that there are two elements $e_{1},e_{2}\in D=D_{1}\cap D_{2}$ which are in the same *bucket* of $\sigma_{1}^{h}$ but in different *buckets* of $\sigma_{2}^{h}$. Since *e*_1_ and *e*_2_ belong to the same $\sigma_{1}^{h}$ bucket it follows, by definition, that *pos*_1_(*e*_1_) = *pos*_1_(*e*_2_) and *pos*_2_(*e*_1_) = *pos*_2_(*e*_2_). Hence, *e*_1_ and *e*_2_ are also in the same $\sigma_{2}^{h}$ *bucket*, which contradicts the initial hypothesis and concludes the proof.

**Algorithm 1**: Homogenization

 **Data**: Two bucket orders *π*_1_ and *π*_2_ on domains $D_{1}$ and $D_{2}$ respectively.

 **Result**: $\pi_{1}^{h}$, the homogenized bucket order of *π*_1_ with respect to *π*_2_.

1 **if** $D_{1}\cap D_{2}=\emptyset$ **then return** *an empty bucket order*;

2 Let $B^{1}=\left(B_{1}^{1},\ldots ,B_{|B^{1}|}^{1}\right)$ and $B^{2}=\left(B_{1}^{2},\ldots ,B_{|B^{2}|}^{2}\right)$ be the ordered sequences of buckets of *π*_1_ and *π*_2_ respectively.

 // $\forall e\in D_{2}$, get the position of the bucket containing *e* in $B^{2}$

3 *e*_*to*_*pos*_2_ ← an empty hash table;

4 **for** *i* from 1 to $|B^{2}|$ **do**

5  **foreach** *e* in $B_{i}^{2}$ **do** *e*_*to*_*pos*_2_.*insert*(*key* = *e*, *value* = *i*);

 // homogenize sequentially all buckets of *B*^1^ to build up $\pi_{1}^{h}$

6 $\pi_{1}^{h}\leftarrow$ an empty *bucket* order;

7 **for** *i from* 1 to $|B^{1}|$ **do**

  // harvest the position of $B_{i}^{1}$ elements in *π*_2_.

8  *Ltemp* ← an empty list;

9  **foreach** *e* in $B_{i}^{1}$ **do**

10   **if** *e* ∈ *e*_*to*_*pos*_2_ **then** *Ltemp*.push(*e*,*e*_*to*_*pos*_2_.getValue(*e*));

  // sort *Ltemp* elements (*e*, *pos*_2_) by increasing *pos*_2_.

11  *LtempSort* ← sort_increasing(*Ltemp*);

  // create a new bucket per set of (now consecutive) elements of $B_{i}^{1}$ which are in a same bucket in *π*_2_.

12  *buck*_*pos*_2_ ← *LtempSort*[1].*second*;

13  *Buck* ← a new empty *bucket*;

14  **for** (*e*, *pos*_2_) from *LtempSort*[1] to *LtempSort*[|*LtempSort*|] **do**

15   **if** *buck*_*pos*_2_ = *pos*_2_ **then**

16    *Buck*.add(*e*);

17   **else** // new bucket

18    $\pi_{1}^{h}$.push_back(*Buck*);

19    *Buck* ← new Buck(*e*);

20    *buck*_*pos*_2_ ← *pos*_2_;

  // handle last bucket

21  $\pi_{1}^{h}$.push_back(*Buck*);

22 **return** $\pi_{1}^{h}$;

Before presenting our homogenization algorithm (Algorithm 1), we first give a lemma (proof in S1 Proof) that provides a simple way to homogenize a bucket order *π*_1_ with respect to *π*_2_, when elements in *π*_1_ buckets are ordered according to their bucket positions in *π*_2_.

**Lemma 1**. Let $\pi_{1}=\left(B_{1}^{1},\ldots ,B_{k1}^{1}\right)$ and $\pi_{2}=\left(B_{1}^{2},\ldots ,B_{k2}^{2}\right)$ be two bucket orders on $D_{1}$ and $D_{2}$ respectively. Let $B_{i}^{\prime 1}$ be an ordered restriction of $B_{i}^{1}$ containing only elements of $B_{i}^{1}\cap D_{2}$ organized in increasing order according to their position in *π*_2_, then $\pi_{1}^{h}$, the homogenized version of *π*_1_ with respect to *π*_2_, is obtained from *π*_1_ by splitting each of its $B_{i}^{\prime 1}=\left(e_{i_{1}},\ldots ,e_{i_{|B_{i}^{\prime 1}|}}\right)$ buckets between two consecutive elements $e_{i_{s}}$ and $e_{i_{s+1}}$ if and only if $e_{i_{s}}$ and $e_{i_{s+1}}$ are in different buckets in *π*_2_ $\forall 1\leq s\leq |B_{i}^{\prime 1}|-1$.

The Algorithm 1 homogenizes one bucket order considering a second bucket order. To get both $\pi_{1}^{h}$ and $\pi_{2}^{h}$ it suffices to use the algorithm twice.

**Proposition 1** (Algorithm 1 correction). When called with parameters *π*_1_ and *π*_2_, the Algorithm 1 returns the homogenization $\pi_{1}^{h}$ of *π*_1_ with respect to *π*_2_.

(Proof in S2 Proof)

**Proposition 2** (Time complexity of Algorithm 1). The overall complexity of Algorithm 1 is *O*(*n* log(*n*)) with $n=\max(|D_{1}|,|D_{2}|)$

**Proof**. The most time consuming operations are:

1. Initialization of *e*_*to*_*pos*_2_ dictionary: $O|D_{2}\left|*\log(\right|D_{2}\left|\right)$ (L. 4-5);
2. Creation of the *Ltemp* lists: $O(|D_{1}\left|*\log(\right|D_{2}\left|)\right)$ as each element of $D_{1}$ is added only once to a *Ltemp* list and this addition is made in $O(\log|D_{2}|)$ due to the interrogation of the *e*_*to*_*pos*_2_ dictionary (L. 9-10);
3. Sorting of the *Ltemp* lists: at the *i*^*th*^ iteration the list contains $|B_{i}^{1}|$ elements that can be sorted using Smoothsort [27] in $O(|B_{i}^{1}\left|\log(\right|B_{i}^{1}\left|)\right)=O(|B_{i}^{1}\left|\log(\right|D_{1}\left|)\right)$. The total time complexity of this loop is thus $O(|D_{1}\left|\log(\right|D_{1}\left|)\right)$ (L. 11).

All other instructions are in *O*(1), thus the overall complexity of Algorithm 1 is:

$$O(|D_{2}\left|*\log(\right|D_{2}\left|)+\right|D_{1}\left|*\log(\right|D_{2}\left|)+\right|D_{1}\left|*\log(\right|D_{1}\left|)\right)=O(\max(|D_{1}\left|,\right|D_{2}\left|)*\log(\max(\right|D_{1}\left|,\right|D_{2}\left|)\right)=O\left(n\log\left(n\right)\right)$$.

It is easy to see that Algorithm 1 performs two distinct tasks: sorting elements in *π*_1_ buckets according to their bucket positions in *π*_2_ and then splitting the buckets of *π*_1_. The former is done in *O*(*n* log(*n*)) while the latter is done in *O*(*n*), for two bucket orders of *n* elements. To achieve a linear time complexity for the overall procedure, it is therefore sufficient to provide sorted buckets to Algorithm 1 or to reduce the complexity of the first task. We give in S1 Algo a linear time variant of Algorithm 1, assuming that bucket orders are composed of pointers to elements of the domain as assumed in [28].

It is already known that bucket order comparisons algorithmically behave like total order comparisons [29] once each bucket order has been refined with respect to the other [23]. Our contribution is to propose straightforward algorithms to do this refinement. The two versions of our homogenization procedure are easy to implement, rigorously proven and shown to achieve low time complexity: *O*(*n* log(*n*)) and *O*(*n*) respectively. For the latter, we were inspired by the first two steps of the preprocessing used for computing distances between partial orders [28].

## Homogenization preserves the LC(I)S of two bucket orders

Below we show that we can search for the LC(I)S of bucket orders using their homogenized counterparts without losing any solutions.

**Theorem 1**. Given *π*_1_ a bucket order on $D_{1}$ and *π*_2_ a bucket order on $D_{2}$, the set of the common subsequences of *π*_1_ and *π*_2_ is identical to the set of the common subsequences of their homogenized counterpart $\pi_{1}^{h}$ and $\pi_{2}^{h}$.

**Proof**. subsequence (*π*_1_, *π*_2_) ⊇ subsequence ($\pi_{1}^{h},\pi_{2}^{h}$)

Let *s* ∈ subsequence($\pi_{1}^{h}$,$\pi_{2}^{h}$). Suppose that *s* ∉ subsequence(*π*_1_), as a consequence there are successive elements *s*[*i*] and *s*[*i* + 1] so that $s\left[i+1\right]{≺}_{\pi_{1}}s\left[i\right]$. Hence there exists a bucket *B*_*k*_ ∋ *s*[*i* + 1] preceding a bucket *B*_*l*_ ∋ *s*[*i*] and *pos*_1_(*s*[*i* + 1]) < *pos*_1_(*s*[*i*]) and, by construction, $s\left[i+1\right]{≺}_{\pi_{1}^{h}}s\left[i\right]$, hence a contradiction and *s* ∈ subsequence(*π*_1_). We can show in the same way that *s* ∈ subsequence(*π*_2_), therefore *s* ∈ subsequence(*π*_1_, *π*_2_).

subsequence(*π*_1_, *π*_2_) ⊆ subsequence ($\pi_{1}^{h},\pi_{2}^{h}$)

By contradiction. Let *s* ∈ subsequence(*π*_1_, *π*_2_). Since all elements of *s* are present in both *π*_1_ and *π*_2_ they also are in $D=D_{1}\cap D_{2}$ hence in $\pi_{1}^{h}$. Suppose that *s* is not a subsequence of $\pi_{1}^{h}$, it follows that there are two successive elements of *s*, *s*[*i*] and *s*[*i* + 1], so that $s\left[i+1\right]{≺}_{\pi_{1}^{h}}s\left[i\right]$. As a consequence there should exist in $\pi_{1}^{h}$ a bucket *B*_*k*_ ∋ *s*[*i* + 1] preceding a bucket *B*_*l*_ ∋ *s*[*i*] and either:

- *s*[*i*] and *s*[*i* + 1] are in the same *bucket* in both *π*_1_ and *π*_2_. However in such a case, by construction *s*[*i*] and *s*[*i* + 1] are in the same $\pi_{1}^{h}$ bucket, hence a contradiction.
- *s*[*i*] and *s*[*i* + 1] are in different *π*_1_ buckets and since *s* is a subsequence of *π*_1_, it follows that *pos*_1_(*s*[*i*]) < *pos*_1_(*s*[*i* + 1]). As a consequence, *s*[*i*] belongs, by construction, to a bucket of $\pi_{1}^{h}$ preceding the one containing *s*[*i* + 1], hence a contradiction.
- *s*[*i*] and *s*[*i* + 1] are in the same bucket in *π*_1_ but not in *π*_2_. Since *s* is a subsequence of *π*_2_, *pos*_2_(*s*[*i*]) < *pos*_2_(*s*[*i* + 1]). As a consequence, *s*[*i*] belongs, by construction, to a bucket of $\pi_{1}^{h}$ preceding the one containing *s*[*i* + 1], hence a contradiction.

As all possible cases lead to a contradiction, the initial hypothesis is impossible and *s* ∈ subsequence($\pi_{1}^{h}$). We can show in the same way that *s* ∈ subsequence($\pi_{2}^{h}$) and therefore that *s* is a subsequence of $\pi_{1}^{h}$ and $\pi_{2}^{h}$.

**Theorem 2**. Given *π*_1_ a bucket order on $D_{1}$ and *π*_2_ a bucket order on $D_{2}$, the set of the common induced subsequences of *π*_1_ and *π*_2_ is identical to the set of the common induced subsequences of their homogenized counterpart $\pi_{1}^{h}$ and $\pi_{2}^{h}$.

**Proof**. indSubsequence (*π*_1_, *π*_2_) ⊆ indSubsequence ($\pi_{1}^{h},\pi_{2}^{h}$)

Let *s* ∈indSubsequence(*π*_1_, *π*_2_). First *s* ∈subsequence($\pi_{1}^{h},\pi_{2}^{h}$) (Def. 4 and Theorem 1). Second, by Definition 4, ∀1 < *i* < *j* < *l*: either i) $s\left[i\right]{≺}_{\pi_{1}}s\left[j\right]$ or ii) $s\left[i\right]{≺}_{\pi_{2}}s\left[j\right]$. In case i) *s*[*i*] is in a bucket preceding *s*[*j*] in $\pi_{1}^{h}$. Hence *s*[*i*] and *s*[*j*] satisfy the 2(*a*) condition required for *s* to be in indSubsequence($\pi_{1}^{h},\pi_{2}^{h}$). Similarly, in case ii) *s*[*i*] is in a bucket preceding *s*[*j*] in $\pi_{2}^{h}$. Hence *s*[*i*] and *s*[*j*] satisfy the 2(*b*) condition required for *s* to be in indSubsequence($\pi_{1}^{h},\pi_{2}^{h}$)

indSubsequence (*π*_1_, *π*_2_) ⊇ indSubsequence ($\pi_{1}^{h},\pi_{2}^{h}$)

Let *s* ∈ indSubsequence($\pi_{1}^{h},\pi_{2}^{h}$). First *s* ∈ subsequence(*π*_1_, *π*_2_) (Def. 4 and Theorem 1). Second, by Definition 4, ∀1 < *i* < *j* < *l*: either i) $s\left[i\right]{≺}_{\pi_{1}^{h}}s\left[j\right]$ or ii) $s\left[i\right]{≺}_{\pi_{2}^{h}}s\left[j\right]$. In case i) *s*[*i*] being in a bucket preceding *s*[*j*] in $\pi_{1}^{h}$ implies that either $s\left[i\right]{≺}_{\pi_{1}}s\left[j\right]$ or ($s\left[i\right]{≹}_{\pi_{1}}s\left[j\right]$ and $s\left[i\right]{≺}_{\pi_{2}}s\left[j\right]$). In both cases *s*[*i*] and *s*[*j*] satisfy the 2^*nd*^ condition required for *s* to be in indSubsequence(*π*_1_, *π*_2_). Similarly in case ii) *s*[*i*] being in a bucket preceding *s*[*j*] in $\pi_{2}^{h}$ implies that either $s\left[i\right]{≺}_{\pi_{2}}s\left[j\right]$ or ($s\left[i\right]{≹}_{\pi_{2}}s\left[j\right]$ and $s\left[i\right]{≺}_{\pi_{1}}s\left[j\right]$). In both cases *s*[*i*] and *s*[*j*] satisfy the 2^nd^ condition required for *s* to be in indSubsequence(*π*_1_, *π*_2_).

Theorems 1 and 2 provide a formal proof that searching for the LC(I)S of two bucket orders *π*_1_ and *π*_2_ gives the same result as searching for the LC(I)S of their two homogenized counterparts $\pi_{1}^{h}$ and $\pi_{2}^{h}$.

## Algorithm for LC(I)S of two bucket orders

Note that the number of markers is potentially decreased by the homogenization procedure and that $\pi_{1}^{h}$ and $\pi_{2}^{h}$ have the same (number of) markers and buckets (Property 1). In this section we denote by $|D_{1}|$ and $|D_{2}|$ the number of elements of *π*_1_ and *π*_2_ respectively; by *n* the maximum of those two values; and by *n*_*b*_ and *n*_*h*_ the number of buckets and markers contained in the homogenized orders $\pi_{1}^{h}$ and $\pi_{2}^{h}$. It follows *n*_*b*_ ≤ *n*_*h*_ ≤ *n*.

**Lemma 2**. After homogenization, buckets are either fully identical or share no common element (*Proof*. Direct consequence of Property 1).

**Lemma 3**. The LCIS of $\pi_{1}^{h}$ and $\pi_{2}^{h}$ contain only one element per homogenized bucket (*Proof*. Direct consequence of Definition 4).

It follows from these two lemmas 2 properties:

**Property 2**. Finding a LCIS of *π*_1_ and *π*_2_ is finding a LCS of the bucket sequences of $\pi_{1}^{h}$ and $\pi_{2}^{h}$.

**Property 3**. Finding a LCS of *π*_1_ and *π*_2_ is finding a Heaviest Common Subsequence (HCS) of the bucket sequences of $\pi_{1}^{h}$ and $\pi_{2}^{h}$ where each bucket is weighted by its number of elements.

**Fig 2:**
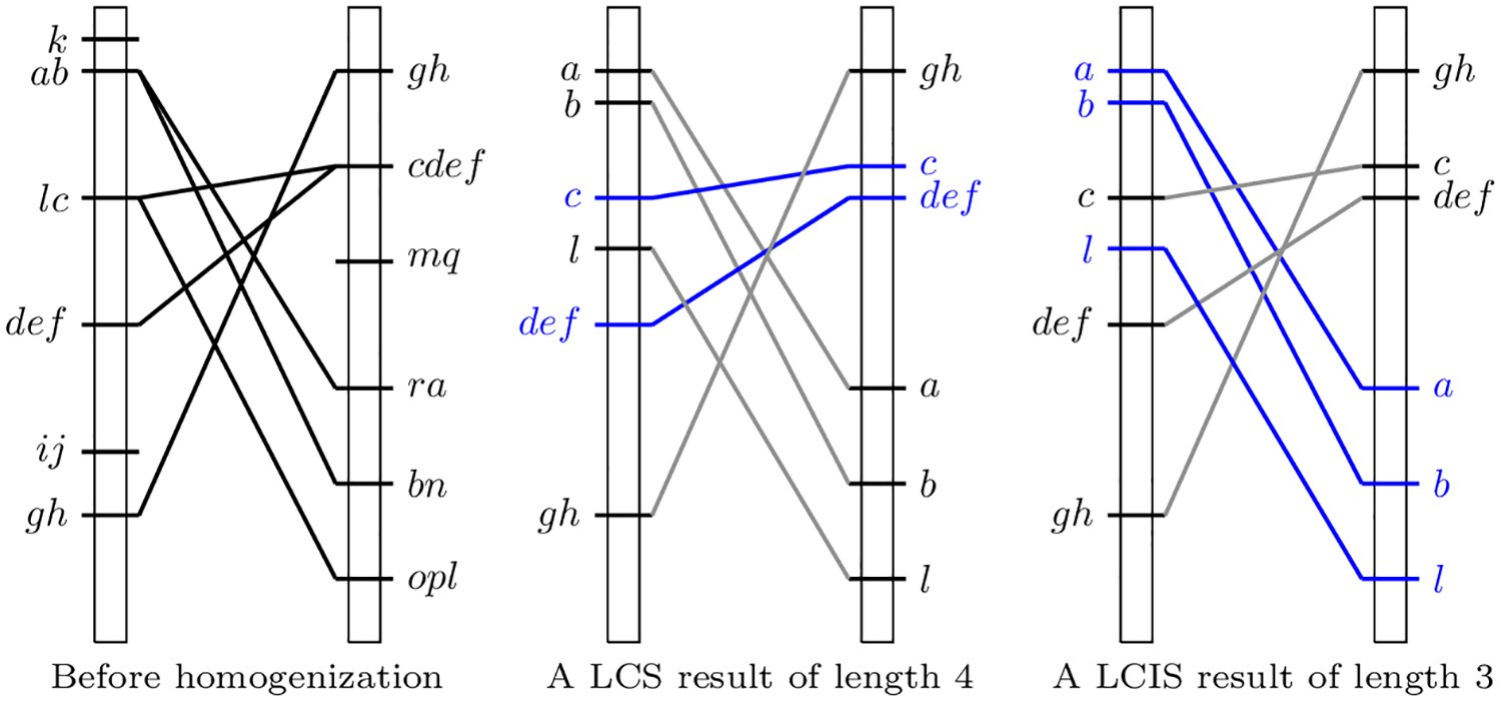
Comparison of two genetic maps before (left) and after (middle and right) homogenization. The blue elements of the scheme in the middle proposes a consensus without conflict corresponding to an LCS, while on the right they show a consensus without conflict corresponding to the LCIS of *π*_1_ = ({*k*}, {*a*, *b*}, {*l*, *c*}, {*d*, *e*, *f*}, {*i*, *j*}, {*g*, *h*}) and *π*_2_ = ({*g*, *h*}, {*c*, *d*, *e*, *f*}, {*m*, *q*}, {*r*, *a*}, {*b*, *n*}, {*o*, *p*, *l*}).

Fig 2 (left) illustrates the comparison of genetic maps *π*_1_ and *π*_2_, as well as the comparison of genetic maps from $\pi_{1}^{h}$ and $\pi_{2}^{h}$ (middle and right) in terms of LC(I)S results (in blue). A subset of non conflicting markers can be seen as a set of non-intersecting edges.

To compute the LC(I)S of two bucket orders *π*_1_ and *π*_2_, we can use the classical quadratic dynamic programming scheme for LCS/HCS [30] on the two sequences of buckets of the homogenized orders $\pi_{1}^{h}$ and $\pi_{2}^{h}$. This is what is done in Algorithm 2. The only subtlety is to manage to recognize identical buckets in constant time. For this, it is sufficient to note that even if the elements of a bucket are incomparable, they are represented in memory by a linear structure. It is then sufficient to choose a total order on $D=D_{1}\cap D_{2}$ (*e.g*., the lexicographic order) and to represent all the buckets of *π*_1_ and *π*_2_ in this order. Testing the equality of two buckets is then equivalent to testing the equality of their first elements (*cf*. Line 9 of Algorithm 2).

Algorithm 2 begins with the homogenization of *π*_1_ and *π*_2_, assuming their buckets are ordered using the same total order, and then classically fills the dynamic programming matrix with the lengths of the LCS or LCIS of $\pi_{1}^{h}$ and $\pi_{2}^{h}$ prefixes, depending on whether the boolean *induced* is false or not. Note that the homogenization (Algorithm 1) does not modify the order of bucket elements if they are already ordered in the same way, so buckets of $\pi_{1}^{h}$ and $\pi_{2}^{h}$ are also ordered according to the same total order as those of *π*_1_ and *π*_2_. It then follows the backtracking procedure that retrieves a LC(I)S.

**Proposition 3**. (Algorithm 2 correction). Given two bucket orders *π*_1_ and *π*_2_ and a boolean *induced*, Algorithm 2 returns a LCS of *π*_1_ and *π*_2_ if *induced* is false, and an LCIS of *π*_1_ and *π*_2_ otherwise.

**Algorithm 2**: LC(I)S

 **Data**: Two bucket orders *π*_1_ and *π*_2_ with bucket elements ordered according to a same total order and a boolean *induced*

 **Result**: One of the LC(I)S of input orders.

 // homogenizing the two bucket orders

1 $\pi_{1}^{h}$ ← Homogenization(*π*_1_,*π*_2_); $\pi_{2}^{h}$ ← Homogenization(*π*_2_,*π*_1_);

2 Let $B^{h_{1}}=\left(B_{1}^{h_{1}},\ldots ,B_{|B^{h_{1}}|}^{h_{1}}\right)$ and $B^{h_{2}}=\left(B_{1}^{h_{2}},\ldots ,B_{|B^{h_{2}}|}^{h_{2}}\right)$ be the ordered sequences of buckets of $\pi_{1}^{h}$ and $\pi_{2}^{h}$ respectively

3 $n_{b}\leftarrow \left|B^{h_{1}}\right|$;                //$|B^{h_{1}}\left|=\right|B^{h_{2}}|$ by Property 1

 // filling the matrix L with the LC(I)S lengths of $\pi_{1}^{h}$ and $\pi_{2}^{h}$ prefixes

4 *L* ← a new matrix of size (*n*_*b*_ + 1) × (*n*_*b*_ + 1);

5 **for** *i* from 0 to *n*_*b*_ **do**

6  *L*[*i*, 0] ← 0; *L*[0, *i*] ← 0;

7 **for** *i* from 1 to *n*_*b*_ **do**

8  **for** *j* from 1 to *n*_*b*_ **do**

9   **if** $B_{i}^{h_{1}}\left[1\right]=B_{j}^{h_{2}}\left[1\right]$ **then**

10    **if** *induced* **then**

11     *L*[*i*, *j*] ← *L*[*i* − 1, *j* − 1] + 1;

12    **else**

13     $L\left[i,j\right]\leftarrow L\left[i-1,j-1\right]+|B_{i}^{h_{1}}|$;

14   **else**

15    *L*[*i*, *j*] ← max(*L*[*i* − 1, *j*], *L*[*i*, *j* − 1]);

 // building a LC(I)S of *π*_1_ and *π*_2_ by backtracking L

16 *τ* ← an empty sequence; *i* ← *n*_*b*_; *j* ← *n*_*b*_;

17 **while** *i* > 0 et *j* > 0 **do**

18  **if** $B_{i}^{h_{1}}\left[1\right]=B_{j}^{h_{2}}\left[1\right]$ **then**

19   **if** *induced* **then**

20    $\tau .push\_front(B_{i}^{h_{1}}\left[1\right])$

21   **else**

22    **foreach** *e* in $B^{h_{1}}$ **do**

23     *τ*.*push*_*front*(*e*)

24   *i* − −; *j* − −;

25  **else if** *L*[*i*, *j* − 1] > *L*[*i* − 1, *j*] **then**

26   *j* − −;

27  **else**

28   *i* − −;

29 **return** *τ*;

**Proof**. The proof is straightforward as Algorithm 2 uses the classical methods [31] to retrieve an HCS of $\pi_{1}^{h}$ and $\pi_{2}^{h}$, giving an LCS of *π*_1_ and *π*_2_ (Property 3), when *induced* is false and an HCS of $\pi_{1}^{h}$ and $\pi_{2}^{h}$, giving an LCIS of *π*_1_ and *π*_2_ (Property 2), otherwise.

Time complexity of Algorithm 2 depends on 3 points:

1. Homogenization is *O*(*n*) or *O*(*n* log(*n*)) depending whether the linear version of the homogenization algorithm is used or not (Line 1);
2. The filling of the matrix is $O(n_{b}^{2})$, with *n*_*b*_ the number of buckets (Lines 4-15);
3. The backtracking procedure is *O*(*n*_*b*_) for retrieving a LCIS or *O*(*n*_*h*_) to retrieve a LCS (Lines 16-28).

The overall time complexity of Algorithm 2, dominated by points 1 and 2, is thus at most $O(n\;\text{log}\left(n\right)+n_{b}^{2})$. In the worst case scenario, where all buckets contain only one marker and all markers are present in both *π*_1_ and *π*_2_, *n*_*b*_ equals *n* and the complexity is *O*(*n*^2^) –just as for the naive solution. In all other cases our solution has a lower time complexity and is faster. The gain in performance increases with the size of the buckets and the number of markers appearing in a single input order.

We give in S2 Algo an alternative version of Algorithm 2 that does not need to assume a total order on D nor similar bucket orderings as it includes bucket order preprocessing (done by Algorithm 1 presented in the following section).

The use of the classical dynamic programming approach has several advantages. Building and storing the full dynamic matrix of intermediary common subsequence lengths allows Algorithm 2 not only to get the length of LC(I)S (stored in the last matrix cell), but also to build a LC(I)S using the backtrack procedure. This also allows, with slight adaptation of the backtracking procedure, to count all the LC(I)S or to return several of them instead of a single one.

To improve time complexity, we can benefit from the LCS algorithmic improvements such as the one of Masek and Paterson [16] that, assuming that the sizes of subsequences are bounded, avoids having to fill the whole dynamic matrix and gives a faster algorithm with *O*(*n* log(*n*) + *n*_*b*_ log(*n*_*b*_)) time. Moreover, if one is only interested in getting the length of the LC(I)S, or getting a sole LC(I)S representative among the possibly numerous ones, a more efficient solution to tackle this problem is to rely on the Longest Increasing Subsequence (LIS). A problem that can be solved in *O*(*n* log(*n*)).

In this section we have shown that we can use the classical LCS approach on bucket orders with the same quadratic time complexity. The advantage of considering bucket orders is that the solution is quadratic on the number of homogenized buckets instead of being quadratic in the number of markers within the input maps. When numerous markers are positioned on the same location, or when the compared map have numerous specific markers, this leads to a drastic improvement in speed.

## Time complexity improvement using LIS

**Fig 3:**
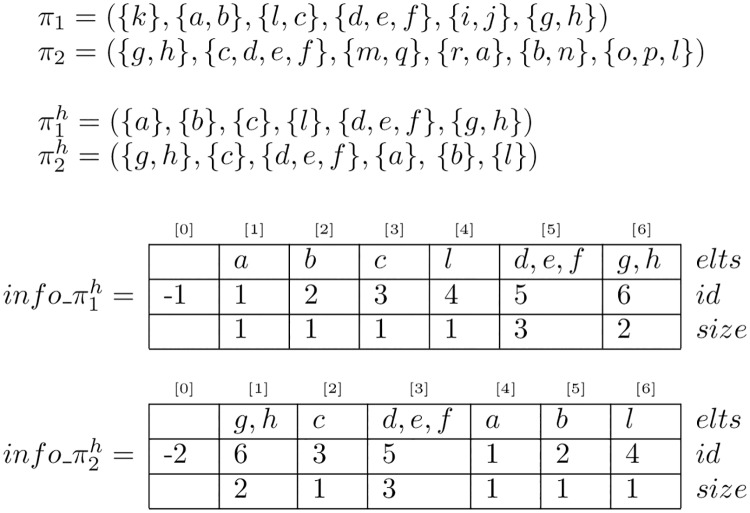
Example of computation of LCIS using LIS with *π*_1_ and *π*_2_. The LIS of the sequence of identifiers from $info\_\pi_{2}^{h}\left[1\right]$, (6, 3, 5, 1, 2, 4), is (1, 2, 4) and gives the LCIS of *π*_1_ and *π*_2_: (*a*, *b*, *l*).

**Fig 4:**
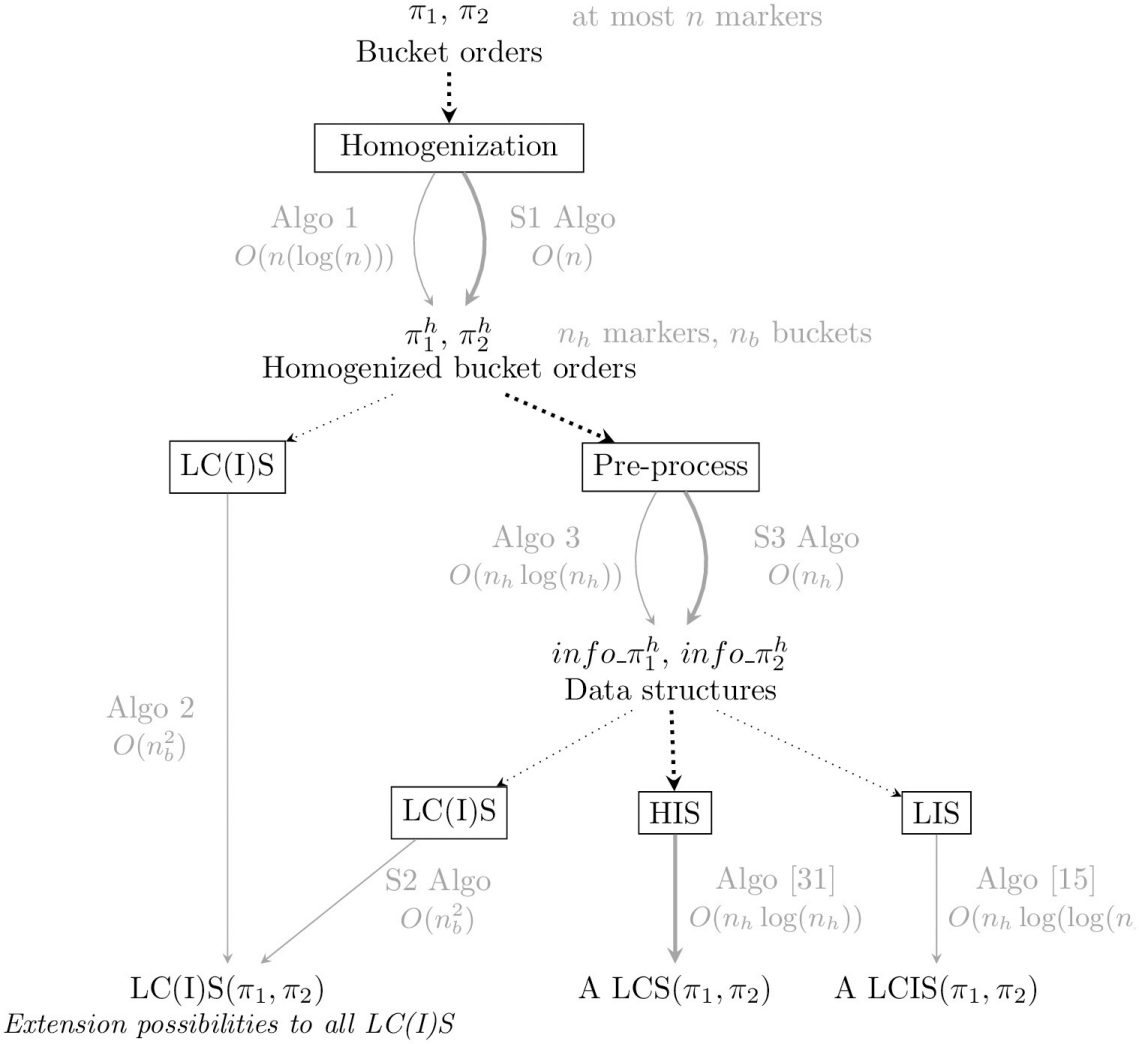
A graph summarizing algorithmic contributions on bucket orders. Each root to tip path of this graph provides a pipeline that chains algorithms to obtain the LCS, the LCIS, or both the LCS and LCIS of 2 input bucket orders *π*_1_ and *π*_2_. The framed nodes of this graph represent the computation steps while the other nodes are the input and/or output of those steps. The algorithms that can be used at each computation step, together with its complexity for this specific task are shown in grey. Note that we count only the time complexity of the specific part of each algorithm. For example, Algorithm 2 calls an algorithm for the Homogenization part, which we don’t count here, as it appears higher on the path. The overall time complexity of a pipeline to get a LCS/LCIS of *π*_1_ and *π*_2_ is the sum of the complexities encountered along the corresponding root to tip path. For example, the time complexity of the pipeline that returns a LCS(*π*_1_, *π*_2_) by chaining S1 and S3 Algo and HIS algo [31] (thick arrow path) is **O**(**n**) + **O**(**n**_**h**_) + **O**(**n**_**h**_ log(**n**_**h**_)) = **O**(**n** log(**n**)).

Once again, to be able to use classical algorithms for the LIS problem we have to carefully pre-treat our bucket orders. We give in this section Algorithm 1 that constructs suitable data structures to encode the necessary information for the LIS computation (see Fig 3 for an example). Note that this preprocessing can also be used combined with Algorithm 1 to compute LCS (see Fig 4). The necessary information elements are for each bucket of each order:

- An identifier (buckets with same identifier are fully identical)
- Its elements
- The number of elements it contains

To test in *O*(1) whether or not a bucket of $\pi_{1}^{h}$ is identical (*i.e*. contains the same elements as) a bucket of $\pi_{2}^{h}$, Algorithm 3 relies on a *id*(.) function that assigns an integer between 1 and $|D_{1}\cup D_{2}|$ to each bucket so that *id*(*B*_*i*_) = *id*(*B*_*j*_) ⇔ *B*_*i*_ = *B*_*j*_.

**Proposition 4**. A unique identifier can be assigned to $\pi_{1}^{h}$ and $\pi_{2}^{h}$ buckets in $O(n\log(|D_{1}\left|)\right)$ and this identifier can be chosen to reflect the bucket position in $B^{h_{1}}$.

**Algorithm 3**: LCS-pre-process

 **Data**: Two bucket orders *π*_1_ and *π*_2_ on domains $D_{1}$ and $D_{2}$ respectively.

 **Result**: Two arrays $info\_\pi_{1}^{h}$ and $info\_\pi_{2}^{h}$ so that $info\_\pi_{1}^{h}\left[i\right]$ (resp $info\_\pi_{2}^{h}\left[i\right]$) contains: the *i*^*th*^ bucket of $\pi_{1}^{h}$ (resp $\pi_{2}^{h}$), the integer identifier of this bucket and the number of elements it contains.

1 $\pi_{1}^{h}$ ← Homogenization(*π*_1_,*π*_2_); $\pi_{2}^{h}$ ← Homogenization(*π*_2_,*π*_1_);

2 Let $B^{h_{1}}=\left(B_{1}^{h_{1}},\ldots ,B_{|B^{h_{1}}|}^{h_{1}}\right)$ and $B^{h_{2}}=\left(B_{1}^{h_{2}},\ldots ,B_{|B^{h_{2}}|}^{h_{2}}\right)$ be the ordered sequences of buckets of $\pi_{1}^{h}$ and $\pi_{2}^{h}$ respectively.

3 *e*_*to*_*buck*_*id*← an empty hash table;

4 $n_{b}\leftarrow \left|B^{h_{1}}\right|$;           //$|B^{h_{1}}\left|=\right|B^{h_{2}}|$ by Property 1

 // Assign bucket id based on their position in $\pi_{1}^{h}$, initiate $info\_\pi_{1}^{h}$

5 $info\_\pi_{1}^{h}$ ← new info array of size *n*_*b*_ + 1;

6 $info\_\pi_{1}^{h}\left[0\right].id\leftarrow -1$;

7 **for** *i from* 1 to *n*_*b*_ **do**

8  $info\_\pi_{1}^{h}\left[i\right].bucket\leftarrow B_{i}^{h_{1}}$;

9  $info\_\pi_{1}^{h}\left[i\right].id\leftarrow i$;

10  $info\_\pi_{1}^{h}\left[i\right].nbElt\leftarrow \left|B_{i}^{h_{1}}\right|$;

11  **foreach** *e* in $B_{i}^{h_{1}}$ **do**

12   *e*_*to*_*buck*_*id*.*add*(*key* = *e*, *value* = *buck*_*id*);

13 $info\_\pi_{2}^{h}$ ← new info array of size *n*_*b*_ + 1;

14 $info\_\pi_{2}^{h}\left[0\right].id\leftarrow -2$;

 // Due to homogenization all elements of a bucket $B_{i}^{h_{2}}$ are in the same $B^{h_{1}}$ bucket, use its first element $B_{i}^{h_{2}}\left[1\right]$ to get its id

15 **for** *i* from 1 to $|B^{h_{2}}|$ **do**

16  $buck\_id\leftarrow e\_to\_buck\_id.getValue(B_{i}^{h_{2}}\left[1\right])$;

17  $info\_\pi_{2}^{h}\left[buck\_id\right].bucket\leftarrow B_{i}^{h_{2}}$;

18  $info\_\pi_{2}^{h}\left[buck\_id\right].id\leftarrow buck\_id$;

19  $info\_\pi_{2}^{h}\left[buck\_id\right].nbElt\leftarrow \left|B_{2}^{h_{1}}\right|$;

20 **return** ($info\_\pi_{1}^{h}$, $info\_\pi_{2}^{h}$);

**Proof**. The procedure is described in Algorithm 3. The first loop iterates over all buckets of $B^{h_{1}}$, initiates $info\_\pi_{1}^{h}$ and saves, for each element, the identifier of the bucket it belongs to in a hash table. This is done in time $O(|D_{1}\left|\log(\right|D_{1}\left|)\right)$. The second loop iterates over all buckets of $B^{h_{2}}$ and for each bucket uses its first element to query the hash table containing the bucket identifier associated to each element. This is done in time $O(|D_{2}\left|\log(\right|D_{1}\left|)\right)$. Hence the overall complexity of $O(\max(|D_{1}\left|,\right|D_{2}\left|)\log(\right|D_{1}\left|\right)$, which is quite similar to *O*(*n* log(*n*)).

Once the two data structures $info\_\pi_{1}^{h}$ and $info\_\pi_{2}^{h}$ are computed by the application of Algorithm 3 on *π*_1_ and *π*_2_, it follows the 2 properties:

**Property 4**. Finding a LCIS of *π*_1_ and *π*_2_ is finding a LIS of the sequence of bucket identifiers stored in $info\_\pi_{2}^{h}$;

**Property 5**. Finding a LCS of *π*_1_ and *π*_2_ is finding, in $info\_\pi_{2}^{h}$, a Heaviest Increasing Subsequence (HIS) of the sequence of bucket identifiers where the elements are weighted by the corresponding bucket size.

Hence, we can obtain an LC(I)S of *π*_1_ and *π*_2_ in *O*(*n* log(*n*) time by using Algorithm 3 followed by either the *O*(*n* log(log(*n*))) LIS algorithm by [15], as our homogenized orders can be consider as permutations, (for LCIS) or by the *O*(*n* log(*n*)) Jacobson and Vo’s algorithm for HIS [31] (for LCS).

We give in S3 Algo a linear time variant of Algorithm 3, assuming that bucket orders are composed of pointers to elements of the domain.

To conclude the methodological part of this article, we present in Fig 4 a graph that sums up our whole contribution in term of algorithms on bucket orders.

## Application to genetic map visual comparison

**Fig 5:**
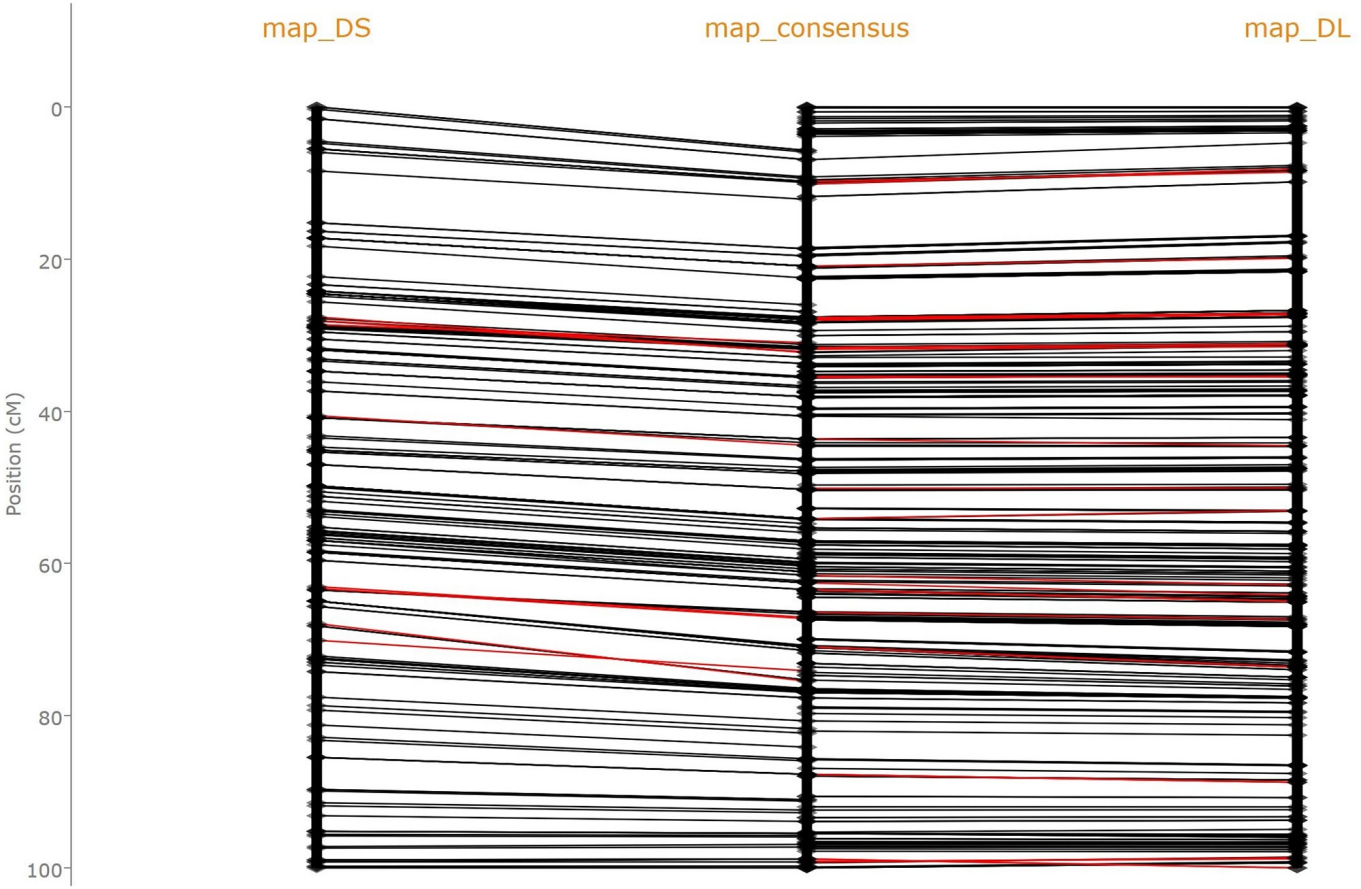
Screenshot of a comparison of three genetic maps of the 3A chromosome of durum wheat displayed by the *Genetic Map Comparator*. The map_DS (right) and map_DL (left) were obtained using different durum wheat progenitors; map_consensus (middle) is the consensus of those two maps as proposed by [32]. For this comparison, two LCS have been computed: the LCS of map_DS and map_consensus and the LCS of map_DL and map_consensus. Markers present in two adjacent maps are then connected by black, or red, edges depending on whether they are, or not, part of their LCS.

Two high density durum wheat genetic maps, each made of thousands of markers, were obtained thanks to high throughput genotyping of the offsprings of two pairs of progenitors: Dic2xLoyd (map_DL) and Dic2xSilur (map_DS) using specific allelic capture and high throughput sequencing [32]. A practical application of finding LC(I)S is illustrated in Fig 5, which is a screenshot of the *Genetic Map Comparator* [12] (http://www.agap-sunshine.inra.fr/genmapcomp/) when used to compare those two durum wheat maps together with their consensus. This visual representation confirms that the maps are highly congruent. Their discrepancies in chromosome 3A, highlighted by the red edges on Fig 5, are circumvent to few regions that could result from small chromosomal rearrangements in those regions between Loyd and Silur progenitors.

**Fig 6:**
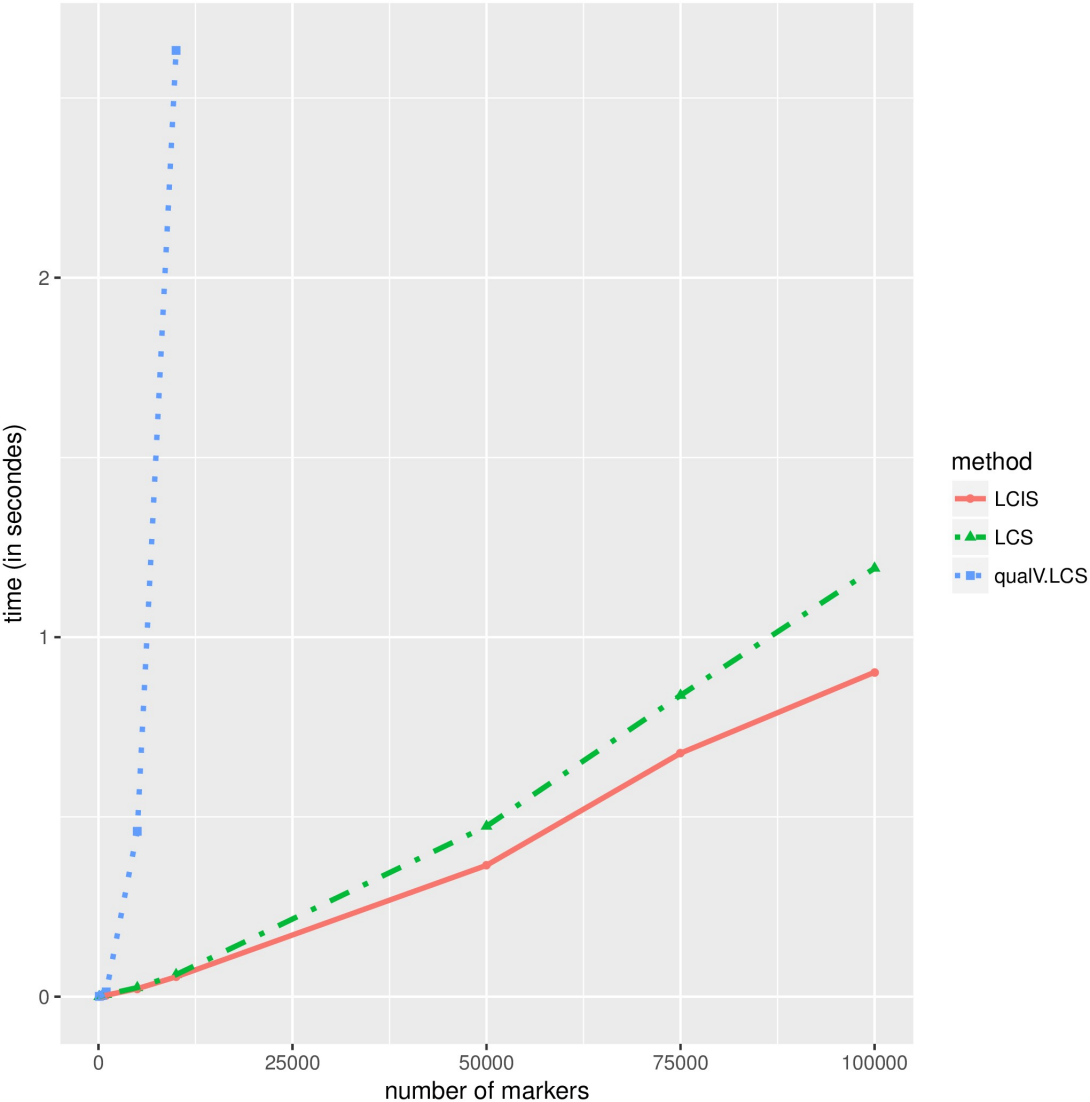
Comparison of computation times in seconds (Y axis) needed to compute LC(I)S for an increasing number of markers (X axis). Three methods are compared: our bucket dedicated methods to compute 1/ LCS (dashed green line) or 2/ LCIS (plain red line) and 3/ a brute force solution ignoring bucket order specificities and relying on the LCS function of the qualV package (blue dotted line). This latter solution is unable to handle large dataset, and crashes the Rstudio environment when called with the 50000 marker dataset whereas our solutions easily handle much larger datasets in seconds.

The Genetic Map Comparator is an R Shiny application made to facilitate genetic map comparisons. One of the challenges for such a tool is to visually emphasize the collinear markers on the two adjacent maps, as well as the breakpoints. This can be done by identifying (a minimal set of) crossing edges and coloring them differently; which can be done by identifying the minimal subset of markers that should be removed to avoid crossing edges. When considering maps as partial orders, corresponding to Directed Acyclic Graph (DAG), the problem is related to the Minimum Breakpoint Linearization problem, which is known to be NP-hard [33]. The Genetic Map Comparator authors’ tackle this problem by using a brute force heuristic to identify congruent markers using the following two-step approach: 1/ for each map a total order is built by tie breaking markers using their position in the other map and, as a last resort, the marker name (a procedure similar to the first step of our homogenization procedure) 2/ the LCS of those two fully ordered sequences of markers is computed using the standard LCS algorithm implemented in the qualV R package [7]. Which turns out to be an exact (but computationally non optimal) solution for the LCS problem of the input bucket orders (this is now obvious thanks to the results provided in this paper). A much more efficient solution is to use the bucket map model and dedicated LC(I)S algorithms described in this paper. As the two compared solutions are guaranteed to return an optimal LCS, the solutions proposed by the two approaches are equally good and the only difference between these methods is the time they need to return the searched LCS. To emphasize the speed up brought about by our solution, we simulate pairs of bucket orders containing 100, 500, 1000, 5000, 10000, 50000, 75000 and 100000 markers. The first order of each pair is obtained by randomly assigning its *n* markers to *n*/10 buckets, while the second order is obtained by swapping 10% of the buckets and moving randomly 10% of the markers (the simulation script is available on LCSLCIS github repository). On the laptop used to conduct the tests (intel i7-6600U, 16Gb RAM), our LC(I)S solution can easily handle datasets of 100,000 markers in seconds whereas the qualV LCS implementation is unable to handle datasets containing 50,000 markers. Both solutions are extremely fast for very small datasets, but the speed difference rapidly increases with the number of markers (Fig 6).

Moreover our formalization of the problem sheds light on the fact that two equally meaningful formulations of this problem exist depending on whether we weight crossing edges by the number of pairs of markers they link (LCS) or not (LCIS) and both problems can be solved efficiently in the special case of bucket orders.

## Conclusion

In this article, we are interested in the problem of comparing two genetic maps by finding their similarities and their differences. For that, we chose to use their LCS which is their largest set of collinear markers. We proposed a new modeling for genetic maps: bucket orders, a precise mathematical object that is able to encode uncertainties about marker positions in maps, while retaining relative position information. We have stated two simple problems: the classical LCS problem adapted to bucket orders and the LCIS problem that prevents the possible random ordering of markers (non comparable in both input orders) observed in the LCS. For each of these problems, we have proposed algorithms that are simple to program, efficient in computation time and rigorously proven. These algorithmic improvements are especially relevant for genetic maps built from SNPs observed in segregating populations where numerous markers are often in total linkage disequilibrium and placed at the exact same position/bucket along the genetic map. These algorithms are implemented in the R package, named LCSLCIS, ready to replace an already existing slower routine used to accomplish the exact same task. Finally, we have illustrated the effectiveness of our approach by applying it to the visual comparison of genetic maps.

The main contribution of the present work is hence twofold. First it provides a theoretical framework when considering genetic maps as bucket orders including a formal definition of i) the LC(I)S for two bucket orders ii) the homogenization of two bucket orders and iii) proof that the LC(I)S is unchanged by the homogenization procedure. Second, it provides a toolkit of simple though efficient algorithms to compute LC(I)S that can be reused by various genetic map related applications. For instance ALLMAPS [6] orient and order sequence scaffolds by minimizing the sum of LCS distance between the considered scaffold organization and some input genetic maps. The LC(I)S procedure introduced here can thus advantageously replace the one used in ALLMAP to efficiently deal with markers located at the same position. The Genetic Map Comparator also relies on an LCS routine to pinpoint incongruent marker positions in different genetic maps and could also benefit from the optimized solution we proposed. Tools which search to build a consensus of several genetic maps, such as MergeMap [34], DAGGER [4] could also benefit from this work. Any genetic map related tool relying on LCS subroutine can safely replace its current LCS subroutine by ours –to do the exact same work but faster– and could thus benefit from a significant speed up while preserving its accuracy. To widen the fields of application, it would be interesting to use these results to design an efficient heuristic able to efficiently search for a genetic map that is the median of several input maps in so far as the LC(I)S related measurements are concerned. Such a heuristic could be used, for example, to construct the backbone of a consensus map efficiently.

## Supporting information

S1 Proof Proof of Lemma 1. (PDF)

S2 Proof Proof of Proposition 1. (PDF)

S1 AlgoLinear homogenization.This algorithm is a linear version of Algorithm 1 assuming that bucket orders are composed of pointers to elements of the domain. This version is inspired by a trick found in [28] to preprocess bucket orders by relabeling the domain D by the integers from 1 to |D|.(PDF)

S2 AlgoLC(I)S from LCS-pre-process.Algorithm S2 Algo is an alternative version of Algorithm 1 that does not need to assume a total order on D nor similar bucket orderings as it relies on the preprocess of Algorithm 1 (or its linear version Algorithm S3 Algo).(PDF)

S3 Algo Linear LCS-pre-process. Algorithm S3 Algo is a linear version of Algorithm 1 assuming that bucket orders are composed of pointers to elements of the domain, using the same trick as Algorithm S1 Algo.
(PDF)
