# Supplementary material for: Efficient algorithms for Longest Common Subsequence of two bucket orders to speed up pairwise genetic map comparison
Source: PLoS One. 2018 Dec 27;13(12):e0208838. doi: 10.1371/journal.pone.0208838 (PMC6320017; doi:10.1371/journal.pone.0208838)
Supplement: S1 Proof — (PDF) [file pone.0208838.s001.pdf]

S1 Proof Proof of Lemma 1.

Lemma 1. *Let* $\pi_1 = (B_1^1, \ldots, B_{k1}^1)$ *and* $\pi_2 = (B_1^2, \ldots, B_{k2}^2)$ *be two bucket orders on* $\mathcal{D}_1$ *and* $\mathcal{D}_2$ *respectively. Let* $B_i'^1$ *be an ordered restriction of* B_i^1 *containing only elements of* $B_i^1 \cap \mathcal{D}_2$ *organized in increasing order according to their position in* π_2*, then* π_1^h*, the homogenized version of* π_1 *with respect to* π_2*, is obtained from* π_1 *by splitting each of its* $B_i'^1 = (e_{i_1}, \ldots, e_{i_{|B_i'^1|}})$ *buckets between two consecutive elements* e_{i_s} *and* $e_{i_{s+1}}$ *if and only if* e_{i_s} *and* $e_{i_{s+1}}$ *are in different buckets in* π_1 $\forall 1 \leq s \leq |B_i^2| - 1$*.*

Proof. Let us demonstrate that π_1^h is the homogenized version of π_1 with respect to π_2.

- **The domain of** π_1^h **is** $\mathcal{D} = \mathcal{D}_1 \cap \mathcal{D}_2$**.** π_1^h is obtained from π_1 by splitting buckets $B_i'^1$, $\forall 1 \leq s \leq |B_i^2|$. Hence, π_1^h is on $\mathcal{D}$.

- **Elements** e **and** e' **of** $\mathcal{D}$ **are in the same bucket in** π_1^h **if and only if they are in the same bucket in** π_1 **and in** π_2**.**

  $\Leftarrow$ By contradiction. Let us suppose that e and e' are in different buckets in π_1^h. Hence, they are in a bucket of π_1 that has been split somewhere between e and e'. However, a bucket is split only between elements that are not in the same π_2 bucket. As the buckets of π_1 are ordered according to their increasing bucket position in π_2, all elements between e et e' (them including) are in the same bucket of π_2, hence a contradiction.

  $\Rightarrow$ If e et e' are in the same bucket of π_1^h, then by construction they are in the same bucket in π_1. Suppose that e and e' are in different buckets in π_2. To find themselves in the same bucket of π_1^h, the bucket of π_1 which contain them has not been split between e and e'. In other words, in this bucket, all elements between e ans e' are in the same bucket in π_2, hence a contradiction.

- $\forall e, e' \in \mathcal{D}$**,** $e \prec_{\pi_1^h} e' \Leftrightarrow e \prec_{\pi_1} e'$ **or** $(e \not\succ\!\!\!\prec_{\pi_1} e'$ **and** $e \prec_{\pi_2} e')$

  $\Leftarrow$ If e is in a bucket which is before the bucket containing e' in π_1, then by construction, the bucket of π_1^h containing e is before the bucket of π_1^h containing e'.

  If e and e' are in the same bucket B of π_1 but e is in a bucket before the bucket containing e' in π_2, then e is before e' in B (because buckets of π_1 are ordered according to their increasing bucket position in π_2). Let e'' be the first element following e in B which is so that e and e' are not in the same bucket in π_2 (such an element always exists since it could be e' itself). The bucket B is then split between e'' and its predecessor, and e is in a bucket before the one containing e' in π_1^h.

  $\Rightarrow$ If e is in a bucket before the one containing e' in π_1^h, then either they are in different buckets in π_1 (with the one containing e before the one containing e'), or they are in the same bucket B of π_1 (with e before e' in

B) which have been split between e and e'. So there is in B an element e'' (which can be e') between e and e' so that e'' is not in the same bucket of π_2 as its direct predecessor in B. Hence, e is in a bucket before the one containing e' in π_2.

$\square$
