# Supplementary material for: Efficient algorithms for Longest Common Subsequence of two bucket orders to speed up pairwise genetic map comparison
Source: PLoS One. 2018 Dec 27;13(12):e0208838. doi: 10.1371/journal.pone.0208838 (PMC6320017; doi:10.1371/journal.pone.0208838)
Supplement: S2 Proof — (PDF) [file pone.0208838.s002.pdf]

**S2 Proof   Proof of Proposition 1.**

**Proposition 1** (Algorithm 1 correction)**.** *When call with parameters* $\pi_1$ *and* $\pi_2$*, the Algorithm 1 returns the homogenization* $\pi_1^h$ *of* $\pi_1$ *with respect to* $\pi_2$*.*

*Proof.* **At each end of the $i^{th}$ loop revolution of Line 7, the** *LtempSort* **list contains exactly the elements of the $i^{th}$ bucket of $\pi_1$, ordered according to their increasing bucket positions in $\pi_2$.**

Lines 4 and 5 fill the hash table $e\_to\_pos_2$ which contains, for each element $e$ in $\pi_2$, the couple $(e, i)$ if and only if element $e$ is in the $i^{th}$ bucket of $\pi_2$.

Line 7 browses buckets $B_i^1$ of $\pi_1$, from $B_1^1$ to $B_{|\mathcal{B}^1|}^1$, and for each element $e$ of $B_i^1$, Lines 9 and 10 add the couple $(e, e\_to\_pos_2.\text{getValue}(e))$ in the *Ltemp* list. Then, Line 11 creates the *LtempSort* list, which contains exactly couples of *Ltemp* ordered according to their second positions, *i.e.*, according to their increasing bucket positions in $\pi_2$.

**When buckets of $\pi_1$ are so ordered, Lemma 1 ensures that $\pi_1^h$ is obtained from $\pi_1$ by splitting each of its buckets between two consecutive elements $e$ and $e'$ if and only if they are in different buckets of $\pi_2$. That is what Lines 12 to 21 do.**

The **"for"** loop of Line 14 browses the ordered *LtempSort* list, at the $i^{th}$ step it browses all the elements of the $i^{th}$ bucket of $\pi_1$, in the order of their increasing bucket positions in $\pi_2$. Line 16 adds element $e$ in the bucket *Buck* in construction if and only if $e$ is the first element of *LtempSort* to be browsed, or if $e$ is in the same bucket of $\pi_2$ as its immediate predecessor (*i.e.*, if $buck\_pos_2 = pos_2$). Otherwise, the bucket *Buck* in construction at the previous step is inserted at the end of $\pi_1^h$ (Line 18), and *Buck* is reset to $e$ (Line 19). □
