# Supplementary material for: Efficient algorithms for Longest Common Subsequence of two bucket orders to speed up pairwise genetic map comparison
Source: PLoS One. 2018 Dec 27;13(12):e0208838. doi: 10.1371/journal.pone.0208838 (PMC6320017; doi:10.1371/journal.pone.0208838)
Supplement: S1 Algo — This algorithm is a linear version of Algorithm 1 assuming that bucket orders are composed of pointers to elements of the domain. This version is inspired by a trick found in [28] to preprocess bucket orders by relabeling the domain D by the integers from 1 to |D|. (PDF) [file pone.0208838.s003.pdf]

S1 Algo. Linear homogenization. This algorithm is a linear version of Algorithm 1 assuming that bucket orders are composed of pointers to elements of the domain. This version is inspired by a trick found in [Bansal *et al.*, 09] to preprocess bucket orders by relabeling the domain $\mathcal{D}$ by the integers from 1 to $|\mathcal{D}|$.

Algorithm S1 Algo: HOMOGENIZATION (LINEAR, ASSUMING ORDERS CONTAIN POINTER TO ELEMENTS INSTEAD OF ELEMENTS)

Data: Two bucket orders π_1 and π_2 on domains $\mathcal{D}_1$ and $\mathcal{D}_2$ respectively.

Result: π_1^h, the homogenized bucket order of π_1 with respect to π_2.

if $\mathcal{D}_1 \cap \mathcal{D}_2 = \emptyset$ **then return** *an empty bucket order*;
Let $\mathcal{B}^1 = (B_1^1, \dots, B_{|\mathcal{B}^1|}^1)$ and $\mathcal{B}^2 = (B_1^2, \dots, B_{|\mathcal{B}^2|}^2)$ be the ordered sequences of buckets of π_1 and π_2 respectively.
// $\forall e \in \mathcal{D}_1$, get the position of the bucket containing e in $\mathcal{B}^1$ if it exists -1 otherwise
foreach e *in* $\mathcal{D}_2$ **do** $e.pos_1 = -1$;
for i *from* 1 *to* $|\mathcal{B}^1|$ **do**
    foreach e *in* B_i^1 **do** $e.pos_1 = i$;
// create a sorted version of $\mathcal{B}^1$ where bucket contents are sorted based on their position in $\mathcal{B}^2$
Let $\mathcal{B}^{sort1} \leftarrow$ an array of $|\mathcal{B}^1|$ empty list $(B_1^{s1}, \dots, B_{|\mathcal{B}^1|}^{s1})$
for i *from* 1 *to* $|\mathcal{B}^2|$ **do**
    foreach e *in* B_i^2 **do**
        if $(e.pos1 > -1)$ **then** $B^{sort1}[e.pos1].push\_back(e, i)$;
// homogenize sequentially all buckets of B^1 to build up π_1^h
$\pi_1^h \leftarrow$ an empty *bucket* order;
for i *from* 1 *to* $|\mathcal{B}^{sort1}|$ **do**
    $LtempSort \leftarrow \mathcal{B}^{sort1}[i]$;
    // create a new bucket per set of (now consecutive) elements of B_i^1 which are in a same bucket in π_2.
    $buck\_pos_2 \leftarrow LtempSort[1].second$;
    $Buck \leftarrow$ a new empty *bucket*;
    for (e, pos_2) *from* $LtempSort[1]$ *to* $LtempSort[|LtempSort|]$ **do**
        if $buck\_pos_2 = pos_2$ **then**
            $Buck$.add(e);
        else // new bucket
            π_1^h.push_back$(Buck)$;
            $Buck \leftarrow$ new Buck(e);
            $buck\_pos_2 \leftarrow pos_2$;
    // handle last bucket
    π_1^h.push_back$(Buck)$;
return π_1^h;
