# Supplementary material for: Efficient algorithms for Longest Common Subsequence of two bucket orders to speed up pairwise genetic map comparison
Source: PLoS One. 2018 Dec 27;13(12):e0208838. doi: 10.1371/journal.pone.0208838 (PMC6320017; doi:10.1371/journal.pone.0208838)
Supplement: S2 Algo — Algorithm S2 Algo is an alternative version of Algorithm 1 that does not need to assume a total order on D nor similar bucket orderings as it relies on the preprocess of Algorithm 1 (or its linear version Algorithm S3 Algo). (PDF) [file pone.0208838.s004.pdf]

**S2 Algo. LC(I)S from LCS-pre-process.** Algorithm S2 Algo is an alternative version of Algorithm 2 that does not need to assume a total order on $\mathcal{D}$ nor similar bucket orderings as it relies on the preprocess of Algorithm 3 (or its linear version Algorithm S3 Algo).

**Algorithm S2 Algo:** LC(I)S FROM LCS-PRE-PROCESS

**Data:** Two bucket orders $\pi_1$ and $\pi_2$ on domain $\mathcal{D}_1$ and $\mathcal{D}_2$ respectively.
A boolean *induced* indicating if we are searching for an induced subsequence or not.
**Result:** One of the LC(I)S of input orders.

$(info\_\pi_1^h, info\_\pi_2^h) \leftarrow$ LCS-pre-process$(\pi_1, \pi_2)$;

$n_b \leftarrow |info\_\pi_1^h|$;

`// filling the matrix L with the LC(I)S lengths of` $\pi_1^h$ `and` $\pi_2^h$ `prefixes`
$L \leftarrow$ a new matrix of size $((n_b + 1) \times (n_b + 1))$;
**for** *i from* 0 *to* $n_b$ **do**
    $L[i, 0] \leftarrow 0$;
    $L[0, i] \leftarrow 0$;

**for** *i from* 1 *to* $n_b$ **do**
    **for** *j from* 1 *to* $n_b$ **do**
        **if** $info\_\pi_1^h[i].id = info\_\pi_2^h[j].id$ **then**
            **if** *induced* **then**
                $L[i, j] \leftarrow L[i-1, j-1] + 1$;
            **else**
                $L[i, j] \leftarrow L[i-1, j-1] + info\_\pi_1^h[i].nbElt$;
        **else**
            $L[i, j] \leftarrow \max(L[i-1, j], L[i, j-1])$;

`// building a LC(I)S of` $\pi_1$ `and` $\pi_2$ `by backtracking L`
$\tau \leftarrow$ an empty sequence ; $i \leftarrow n_b$ ; $j \leftarrow n_b$ ;
**while** $i > 0$ *et* $j > 0$ **do**
    **if** $info\_\pi_1^h[i].id = info\_\pi_2^h[j].id$ **then**
        **if** *induced* **then**
            $\tau.push\_front(info\_\pi_1^h.bucket[1])$
        **else**
            **foreach** *e in* $info\_\pi_1^h.bucket$ **do**
                $\tau.push\_front(e)$
        $i--$; $j--$ ;
    **else if** $L[i, j-1] > L[i-1, j]$ **then**
        $j--$;
    **else**
        $i--$;

**return** $\tau$;
