# Supplementary material for: Efficient algorithms for Longest Common Subsequence of two bucket orders to speed up pairwise genetic map comparison
Source: PLoS One. 2018 Dec 27;13(12):e0208838. doi: 10.1371/journal.pone.0208838 (PMC6320017; doi:10.1371/journal.pone.0208838)
Supplement: S3 Algo — Algorithm S3 Algo is a linear version of Algorithm 1 assuming that bucket orders are composed of pointers to elements of the domain, using the same trick as Algorithm S1 Algo. (PDF) [file pone.0208838.s005.pdf]

S3 Algo. Linear LCS-pre-process. Algorithm S3 Algo is a linear version of Algorithm 3 assuming that bucket orders are composed of pointers to elements of the domain, using the same trick as Algorithm S1 Algo.

Algorithm S3 Algo: LCS-PRE-PROCESS (LINEAR, ASSUMING ORDERS CONTAIN POINTER TO ELEMENTS INSTEAD OF ELEMENTS)

Data: Two bucket orders π_1 and π_2 on domains $\mathcal{D}_1$ and $\mathcal{D}_2$ respectively.

Result: Two arrays $info\_\pi_1^h$ and $info\_\pi_2^h$ so that $info\_\pi_1^h[i]$ (resp $info\_\pi_2^h[i]$) contains: the ith bucket of π_1^h (resp π_2^h), the integer identifier of this bucket and the number of elements it contains.

$\pi_1^h \leftarrow$ Homogenization(π_1,π_2); $\pi_2^h \leftarrow$ Homogenization(π_2,π_1);
Let $\mathcal{B}^{h_1} = (B_1^{h_1}, \ldots, B_{|\mathcal{B}^{h_1}|}^{h_1})$ and $\mathcal{B}^{h_2} = (B_1^{h_2}, \ldots, B_{|\mathcal{B}^{h_2}|}^{h_2})$ be the ordered sequences of buckets of π_1^h and π_2^h respectively.
$n_b \leftarrow |\mathcal{B}^{h_1}|$; $//|\mathcal{B}^{h_1}| = |\mathcal{B}^{h_2}|$ by Property 1
`// Assign bucket identifier based on their position in` π_1^h`, initiate` $info\_pi_1^h$
$info\_pi_1^h \leftarrow$ new info array of size $n_b + 1$;
$info\_pi_1^h[0].id \leftarrow -1$;
$buck\_id \leftarrow 1$;
for *i from* 1 *to* $|\mathcal{B}^{h_1}|$ **do**
    $info\_\pi_1^h[buck\_id].bucket \leftarrow B_i^{h_1}$;
    $info\_\pi_1^h[buck\_id].id \leftarrow buck\_id$;
    $info\_\pi_1^h[buck\_id].nbElt \leftarrow |B_i^{h_1}|$;
    foreach *e in* $B_i^{h_1}$ **do**
        $e.buck\_id\_h1 \leftarrow buck\_id$;
    $buck\_id \leftarrow buck\_id + 1$;

$info\_pi_2^h \leftarrow$ new info array of size $n_b + 1$;
$info\_pi_2^h[0].id \leftarrow -2$;
`// Due to homogenization all elements of a bucket` $B_i^{h_2}$ `are in the same` $\mathcal{B}^{h_1}$ `bucket, use its first element` $B_i^{h_2}[1]$ `to get its id`
for *i from* 1 *to* n_b **do**
    $buck\_id \leftarrow (B_i^{h_2}[1]).buck\_id\_h1$;
    $info\_\pi_2^h[buck\_id].bucket \leftarrow B_i^{h_2}$;
    $info\_\pi_2^h[buck\_id].id \leftarrow buck\_id$;
    $info\_\pi_2^h[buck\_id].nbElt \leftarrow |B_2^{h_1}|$;

return ($info\_\pi_1^h$, $info\_\pi_2^h$);
